# Mutational Analysis at Intersubunit Interfaces of an Anionic Glutamate Receptor Reveals a Key Interaction Important for Channel Gating by Ivermectin

**DOI:** 10.3389/fnmol.2017.00092

**Published:** 2017-04-06

**Authors:** Nurit Degani-Katzav, Revital Gortler, Marina Weissman, Yoav Paas

**Affiliations:** Laboratory of Ion Channels, The Mina and Everard Goodman Faculty of Life Sciences, Institute of Nanotechnology and Advanced Materials, Bar-Ilan UniversityRamat Gan, Israel

**Keywords:** Cys-loop receptors, GluCls, ligand-gated ion channels, ivermectin, parasitic nematodes

## Abstract

The broad-spectrum anthelmintic drug ivermectin (IVM) activates and stabilizes an open-channel conformation of invertebrate chloride-selective glutamate receptors (GluClRs), thereby causing a continuous inflow of chloride ions and sustained membrane hyperpolarization. These effects suppress nervous impulses and vital physiological processes in parasitic nematodes. The GluClRs are pentamers. Homopentameric receptors assembled from the *Caenorhabditis elegans (C. elegans)* GluClα (GLC-1) subunit can inherently respond to IVM but not to glutamate (the neurotransmitter). In contrast, heteromeric GluClα/β (GLC-1/GLC-2) assemblies respond to both ligands, independently of each other. Glutamate and IVM bind at the interface between adjacent subunits, far away from each other; glutamate in the extracellular ligand-binding domain, and IVM in the ion-channel pore periphery. To understand the importance of putative intersubunit contacts located outside the glutamate and IVM binding sites, we introduced mutations at intersubunit interfaces, between these two binding-site types. Then, we determined the effect of these mutations on the activation of the heteromeric mutant receptors by glutamate and IVM. Amongst these mutations, we characterized an α-subunit point mutation located close to the putative IVM-binding pocket, in the extracellular end of the first transmembrane helix (M1). This mutation (αF276A) moderately reduced the sensitivity of the heteromeric GluClαF276A/βWT receptor to glutamate, and slightly decreased the receptor subunits’ cooperativity in response to glutamate. In contrast, the αF276A mutation drastically reduced the sensitivity of the receptor to IVM and significantly increased the receptor subunits’ cooperativity in response to IVM. We suggest that this mutation reduces the efficacy of channel gating, and impairs the integrity of the IVM-binding pocket, likely by disrupting important interactions between the tip of M1 and the M2-M3 loop of an adjacent subunit. We hypothesize that this physical contact between M1 and the M2-M3 loop tunes the relative orientation of the ion-channel transmembrane helices M1, M2 and M3 to optimize pore opening. Interestingly, pre-exposure of the GluClαF276A/βWT mutant receptor to subthreshold IVM concentration recovered the receptor sensitivity to glutamate. We infer that IVM likely retained its positive modulation activity by constraining the transmembrane helices in a preopen orientation sensitive to glutamate, with no need for the aforementioned disrupted interactions between M1 and the M2-M3 loop.

## Introduction

Chloride-selective glutamate receptors (GluClRs) are pentameric glutamate (Glu)-gated chloride channels unique to invertebrates (Wolstenholme, 2012). These receptors belong to the Cys-loop receptor superfamily of transmembrane oligomers that open an intrinsic ion-channel pore upon binding of neurotransmitters such as, acetylcholine (ACh; Karlin, 2002; Lester et al., 2004; Taylor et al., 2007; Taly et al., 2009; Boulin et al., 2012; Sine, 2012; Chatzidaki and Millar, 2015; Dineley et al., 2015; Stokes et al., 2015; Corradi and Bouzat, 2016), serotonin (Lummis, 2012; Kesters et al., 2013), γ-aminobutyric acid (GABA; Zheleznova et al., 2009; Akk and Steinbach, 2011; Morlock and Czajkowski, 2011; Spurny et al., 2012; Liang and Olsen, 2014; Seljeset et al., 2015) glycine (Gly; Betz et al., 1999; Breitinger and Becker, 2002; Colquhoun and Sivilotti, 2004; Betz and Laube, 2006; Harvey et al., 2008; Sivilotti, 2010; Lynagh and Lynch, 2012b; Schaefer et al., 2013; Langlhofer and Villmann, 2016), histamine (Hardie, 1989; Gisselmann et al., 2002; Zheng et al., 2002) or Glu (Wolstenholme, 2012). GluClRs are specific targets for ivermectin (IVM), a macrocyclic lactone widely used as an anthelmintic drug to treat filarial diseases like onchocerciasis (river blindness), which is caused by *Onchocerca volvulus*, and elephantiasis (lymphatic filariasis) that is caused by *Wuchereria bancrofti, Brugia malayi, Brugia timori* and* Brugia pahangithat*. These diseases afflict hundreds of millions of people worldwide, mainly in equatorial Africa (Crump and Ōmura, 2011; Campbell, 2012). IVM is also broadly used in cattle, swine and pets to kill gastrointestinal roundworms, lungworms, grubs, sucking lice and mange mites (Geary, 2005).

IVM acts as an agonist that keeps the ion-channel pore of the GluClR continuously open (Cully et al., 1994; Etter et al., 1996; Dent et al., 1997; Vassilatis et al., 1997; Li et al., 2002; Slimko et al., 2002). Since the GluClR is chloride selective, IVM causes sustained hyperpolarization across postsynaptic membranes in parasitic nematodes. This long-lasting effect eventually leads to suppression of locomotion (Cook et al., 2006); inhibition of the pharyngeal muscle activity, which interrupts with feeding behavior (Geary et al., 1993; Brownlee et al., 1997; Dent et al., 2000); and interruption of secretion processes in the parasite that are crucial for evading the host immune system (Moreno et al., 2010; reviewed in Geary and Moreno, 2012; Wolstenholme, 2012; Wolstenholme et al., 2016).

Notably, IVM activates and/or potentiates a few vertebrate Cys-loop receptors, like GABA-, and Gly-gated Cl^−^ channels (Williams and Risley, 1982; Olsen and Snowman, 1985; Sigel and Baur, 1987; Krůsek and Zemková, 1994; Adelsberger et al., 2000; Shan et al., 2001; Zheng et al., 2002; Pless and Lynch, 2009b; Lynagh and Lynch, 2012a; Ménez et al., 2012; Wang and Lynch, 2012) and the α7 cationic ACh-gated channel (Krause et al., 1998; Collins and Millar, 2010), though with much higher drug concentrations than in GluClRs. IVM can also activate the P2X ATP-gated ion channel belonging to a different family of ligand-gated ion channels (Khakh et al., 1999; Priel and Silberberg, 2004; Silberberg et al., 2007; Habermacher et al., 2016).

Genes (*glc-1* and *glc-2*) encoding two GluClR homologous subunits, GluClα (GLC-1; also named GluClα1) and GluClβ (GLC-2), were firstly cloned from the non-parasitic nematode *C. elegans* (Cully et al., 1994). Later, additional genes encoding subunits of Glu-gated chloride channels were cloned from *C. elegans* (Yates et al., 2003) and other invertebrates (Lynagh et al., 2015) like, parasitic worms (Delany et al., 1998; Jagannathan et al., 1999; Dufour et al., 2013; Lynagh et al., 2014), insects (Eguchi et al., 2006; Dong et al., 2013; Furutani et al., 2014; Kita et al., 2014; Meyers et al., 2015; Wu et al., 2017), crustaceans (Cornejo et al., 2014), and mollusk (Kehoe et al., 2009). In several cases, a single subunit was found to form a functional homomeric receptor–channel that can be gated by both Glu and IVM independently. For example, the GluClα2 (AVR-15) subunit of *C. elegans* (Dent et al., 1997), the DrosGluCl-α subunit of *Drosophila melanogaster* (Cully et al., 1996), the GluClα2B subunit of *H. contortus* (McCavera et al., 2009), the MdGluClα subunit of *Musca domestica* (Eguchi et al., 2006), the GluCl exon-3 variants of *Bombyx mori* (Furutani et al., 2014), and the AgGluCl-a1 of *Anopheles gambiae* (Meyers et al., 2015). In contrast, when expressed in *Xenopus* oocytes, the *C. elegans* GluClα subunit (GLC-1) forms homomeric receptors that can be activated by IVM but not by Glu, whereas the *C. elegans* GluClβ subunit (GLC-2) forms homomeric receptors that can be activated by Glu but not by IVM (Cully et al., 1994; Vassilatis et al., 1997; Li et al., 2002; Daeffler et al., 2014). On the other hand, a heteromeric GluClR consisting of the *C. elegans* α (GLC-1) and β (GLC-2) subunits can be activated by both Glu and IVM independently (Cully et al., 1994; Etter et al., 1996; Dent et al., 1997; Vassilatis et al., 1997; Li et al., 2002; Slimko et al., 2002).

**Figure 1:**
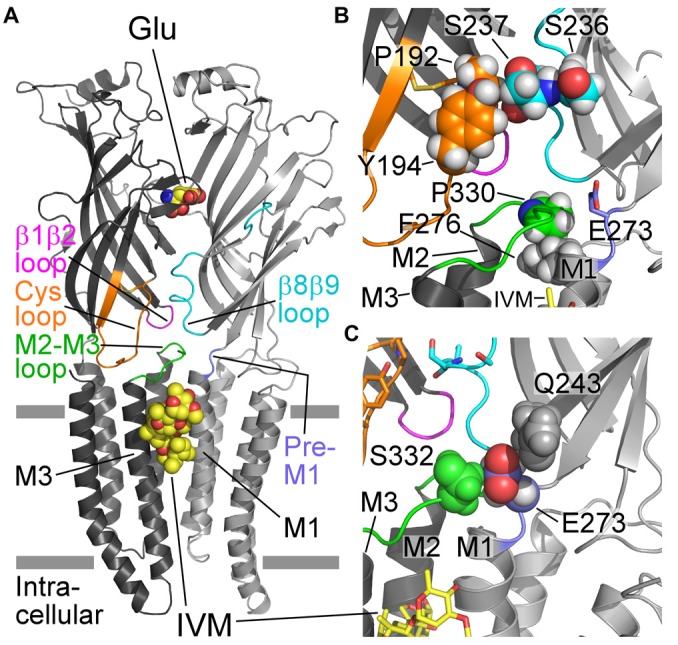
**Structural characteristics of a GluCl receptor. (A)** Two of five subunits of the homopentameric GluClα_cryst_R [Protein Data Bank (PDB) ID code 3RIF] are shown from the side in light and dark gray colors. Wide gray horizontal lines mark the putative membrane borders. The four coupling loops and the pre-M1 linker are colored as shown in **(B,C)**. Glu and ivermectin (IVM) are shown as space-filling models with carbon, oxygen and nitrogen atoms colored in yellow, red and blue, respectively. They are bound at the α/α intersubunit interface far away from each other: Glu in the extracellular ligand-binding domain, and IVM in the upper part of the pore-domain periphery, between M1 (of the light gray subunit) and M3 (of the dark gray subunit). Hydrogen atoms were removed for better viewing. **(B)** Residues relevant to this study are shown as spheres with carbon atoms having the ribbon color, and oxygen, nitrogen and hydrogen atoms in red, blue and white colors, respectively. Only S237 is shown with its backbone atoms. **(C)** E273 (of the pre-M1 linker) is sandwiched between Q243 (gray) and S332 (green) that are located in the β9 strand and the M2-M3 loop of the adjacent subunit, respectively. Only the side chains of the three residues are shown, as space-filling models with their hydrogen atoms. E273 is colored with purple carbons, red oxygens and white hydrogens.

The differential responses of the homomeric *C. elegans* GluClα or GluClβ receptor assemblies suggest that the binding sites for Glu and IVM are uncoupled and, possibly, the conformational changes underlying channel opening by IVM are different from those underlying opening by Glu. Yet, Glu elicits current responses in homomeric *C. elegans* GluClαRs when applied after activation by IVM, indicating that IVM binding to the homomeric *C. elegans* GluClαR induces a conformational change that couples Glu binding at GluCl α/α intersubunit interfaces to the ion-channel gate (Etter et al., 1996). Recently, it was demonstrated that a *C. elegans* heteromeric GluClα/β receptor, whose β subunits were engineered to carry the α-subunit’s Cys and β8β9 loops (Figure 1A), readily responds to Glu, with no need of IVM pre-association (Degani-Katzav et al., 2016). Since the GluCl α and β subunits share the same β1β2-loop sequence, all the coupling loops originating from the ligand-binding domain of this heteromeric mutant receptor (β1β2, Cys and β8β9 loops in each subunit) have the sequence of the α subunit. Hence, it was concluded that these α-subunit’s coupling loops are inherently capable of transducing motions in the Glu-binding site to gating motions in the ion-channel pore (Degani-Katzav et al., 2016). Moreover, a mutation inside the IVM binding pocket (αL279W) increased the sensitivity of the *C. elegans* GluClα/βR to both IVM and Glu (Degani-Katzav et al., 2016), suggesting that the IVM and Glu binding sites in the *C. elegans* GluClα/β heteromeric receptor are allosterically coupled.

**Figure 2:**
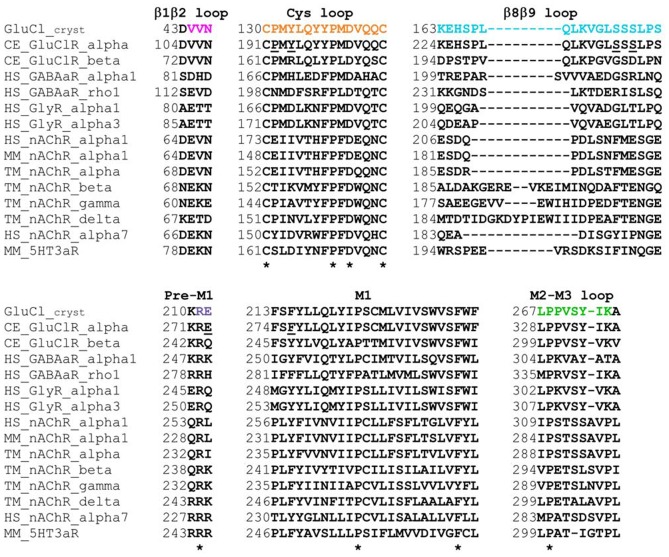
**Sequence alignments of the coupling loops, pre-M1 linker and the first transmembrane segment (M1) in a few Cys-loop receptors.** Colored amino acids in the first row match the colors in Figure 1. Asterisks indicate highly conserved amino acids. GluCl_cryst, a truncated α subunit used for crystallization and 3-D structure determination by X-ray crystallography (PDB ID 3RIF). CE, *Caenorhabditis elegans*; HS, *Homo sapiens*; MM, *Mus musculus* (mouse); TM, *Torpedo marmorata* (Marbled electric ray). UniProt Knowledgebase entry codes: CE_GluClR_alpha, G5EBR3; CE_GluClR_beta, Q17328; HS_GABAaR_alpha1, P14867; HS_GABAaR_rho1, P24046; HS_GlyR_alpha1, P23415; HS_GlyR_alpha3, O75311; HS_nAChR_alpha1, P02708; MM_nAChR_alpha1, P04756; TM_nAChR_alpha, P02711; TM_nAChR_beta, Q6S3I0; TM_nAChR_gamma, Q6S3H9; TM_nAChR_delta, Q6S3H8; HS_nAChR_alpha7, P36544; MM_5HT3aR, P23979.

To understand the importance of putative intersubunit contacts located outside the Glu and IVM binding sites, we introduced mutations at intersubunit interfaces, between these two binding-site types. To this end, we have used the three dimensional (3-D) structure of the GluClα_cryst_ receptor as a guiding tool for substituting residues in the *C. elegans* GluClα (GLC-1) subunit (Figures 1, 2; Table 1) that was co-expressed with the wild type *C. elegans* GluClβ (GLC-2) subunit to form heteromeric receptors. Then, we determined by electrophysiological measurements the effect of these mutations on the activation of the wild type and mutant receptors by glutamate and IVM.

**Table 1:** **Residues discussed in this study**.

GluClα (G5EBR3)^1^	GluClα_cryst_ (3RIF)^2^	Location in GluClα (based on 3RIF)	GluClβ (Q17328)^1^
P192	P131	Cys loop	P162
Y194	Y133	Cys loop	R164
S236	S175	β8β9 loop	G206
S237	S176	β8β9 loop	S207
S238	S177	β8β9 loop	D208
Q243	Q182	β9	I213
E273	E212	Pre-M1	Q244
F276	F215	M1	Y247
P330	P269	M2-M3 loop	P301
S332	S271	M2-M3 loop	S303
I334	I273	M2-M3 loop	V305

*^1^UniProt Knowledgebase entry codes of the full-length subunits used in this study. GluClα, GLC-1; GluClβ, GLC-2. ^2^ID number in the RCSB PDB*.

## Materials and Methods

### Generation of cDNA Constructs

The cDNA encoding the GluClα subunit was a kind gift from Dr. Henry A. Lester (optGluCl alpha EYFP; Plasmid #15104 in Addgene), and it was used to prepare the cDNA of the* C. elegans* GluClαWT subunit (GLC-1; see UniProt entry G5EBR3 for the ORF sequence). The cDNA encoding the *C. elegans* GluClβWT subunit (GLC-2; see UniProt entry Q17328 for the ORF sequence) was prepared by reverse transcription of total *C. elegans* RNA and PCR amplification of the relevant ORF, which was subsequently cloned into a pcDNA3.1 vector. Single or double site-specific mutations were introduced as previously by using the QuikChange site-directed mutagenesis kit (Stratagene; Pittel et al., 2010, 2015). The entire ORF of all mutants was sequenced and subcloned into an original pcDNA3.1 vector.

### Preparation of Cells for Electrophysiological Experiments

Chinese hamster ovary (CHO) cells were cultured as previously (Bar-Lev et al., 2011) in Dulbecco’s Modified Eagle Medium (DMEM) containing 10% fetal calf serum, 2 mM glutamax, 100 Units/ml penicillin G and 100 μg/ml streptomycin, and grown in 5% CO_2_ at 37°C under 90%–95% humidity. The cells were seeded on glass coverslips (13-mm diameter) placed in a 24-multiwell plate. Picrotoxin (5 μM) was added to the cells immediately before transfection, to prevent chloride fluxes through the expressed GluCl channels due to the presence of glutamate in the fetal calf serum. For the expression of GluCl receptors, cells were transiently co-transfected with pcDNA3.1 plasmids containing the ORFs of interest using transit-LT1 transfection reagent (Mirus, Madison, WI, USA), according to the manufacturer’s protocol. The cDNAs encoding the *C. elegans* GluClα (GLC-1) or its mutated versions and the GluClβ (GLC-2) subunits (200–600 ng per well) were used at 1:1 ratio. The pIRES-CD8 plasmid (200 ng per well) was added to enable the visualization of the expressing cells by beads coated with anti-CD8 antibodies (Invitrogen). For high-level expression of the GluClαF276A/βWT mutant receptor in experiments assigned to determine its IVM-EC_50_, we used the X-tremeGENE HP DNA Transfection Reagent (Roche Life Science). Recordings were performed 72–96 h following the transfection.

### Whole-Cell Patch Clamp Recordings

Whole-cell patch clamp recordings were performed as described previously (Bar-Lev et al., 2011) in CHO cells that were prepared as detailed above. The normal external solution (NES) contained (in millimolar): 140 NaCl, 2.8 KCl, 2 CaCl_2_, 2 MgCl_2_, 10 glucose and 10 HEPES, adjusted to pH 7.35 with NaOH (310 mOsm/L). The pipette solution contained (in millimolar): 130 KCl, 4 MgCl_2_, 4 Na_2_ATP, 1 EGTA and 10 HEPES, adjusted to pH 7.35 with KOH (290 mOsm/L). The osmolarity of these solutions was maintained by adding sucrose. The electrode resistance was 6–10 MΩ when filled with the pipette solution. External solutions were applied onto the cell by using the VC-77SP fast-step system (Warner Instruments, Hamden, CT, USA) combined with N_2_ pressure of 3–4 psi to produce laminar flow of the external solution onto the patched cell. All measurements were performed at room temperature. The currents were measured with an Axopatch 200B patch-clamp amplifier (Molecular Devices, Sunnyvale, CA, USA) and a Digidata 1440A interface (Molecular Devices). Acquisition of recording data was performed at 2.5 kHz and recordings were low-pass filtered at 1 kHz, through a four-pole Bessel filter. The pClamp 10 software (Molecular Devices) was used for data acquisition. To establish *I/V* relations, Glu-EC_50_ concentrations were applied for 800 ms. Five-hundred milliseconds after the application started (which is also after the current reached to its peak at −60 mV), the voltage was stepped from −60 mV to −80 mV for 50 ms followed by a 250-ms-long voltage ramp ranging from −80 mV to +80 mV.

### Data Analysis

Dose-response curves were fitted to the data points by a nonlinear regression using the Hill Equation 1,

(1) $$\frac{I}{I_{\max}}\;=\;\frac{1}{1+10^{({\text{logEC}}_{\text{50}}\text{-log}[\text{Glu}])\cdot n_{\text{H}}}}$$

where *I* is the current response, *I*_max_ is the maximal current response, EC_50_ is the agonist effective concentration that elicits 50% of maximal current response, [Glu] is the concentration of glutamate, and *n*_H_ is the Hill coefficient.

### Statistical Analyses

Unless otherwise stated, *P* values correspond to unpaired, two-tailed Student’s *t*-tests. Note that values were rounded to the closest decimal figure; however, the non-rounded numbers were used for the statistical analyses.

## Results

### Activation of GluClR Assemblies by IVM and Glu

All the GluCl receptor assemblies mentioned below originate from the *C. elegans* GluClα (GLC-1) and GluClβ (GLC-2) subunits; so, the species name is avoided hereafter. We have recently shown that CHO cells transfected with the wild type GluClα subunit (αWT) alone display very weak responses to 10 mM Glu but robust responses to 500 nM IVM (Degani-Katzav et al., 2016). It was also shown that CHO cells transfected with the wild type GluClβ subunit (βWT) alone display very weak, rare responses to 10 mM Glu (Degani-Katzav et al., 2016). Others also reported irresponsiveness to Glu in human embryonic kidney (HEK) cells transfected with the wild type GluClβ subunit alone (Slimko et al., 2002; Frazier et al., 2013; Daeffler et al., 2014). Most recently, we succeeded to obtain responses of a few hundred picooamperes in CHO cells transfected with the WT GluClβ subunit alone using the X-tremeGENE HP DNA Transfection Reagent (Roche Life Science); but, in this case, we challenged the cells with 100 mM Glu, and only 4 of 46 cells responded (Degani-Katzav et al., 2017). In contrast, CHO cells co-transfected with both WT GluCl α and β subunits commonly display robust responses to both Glu and IVM (Degani-Katzav et al., 2016). These results are summarized in Table 2. We therefore deduce that common, robust responses to Glu recorded in CHO cells co-transfected with mutant GluClα and wild type GluClβ subunits reflect the function of heteromeric GluClα/βR complexes (Table 2).

**Table 2:** **Macroscopic activation properties of the wild type and mutant GluClα/β receptors**.

GluClR subunit combination	Glu activation properties			Glu (EC_50_ conc.)	IVM (500 nM)
	**EC_50_, mM**	**ANOVA**	***n*_H_^a^**	**Amplitude (nA)**	**Amplitude (nA)^b^**
αWT	ND^c^	–	ND^c^	ND^c^	1.0 ± 0.3 (14)
βWT	ND^c^	–	ND^c^	ND^c^	0.0 ± 0.0 (10)
αWT/βWT	1.5 ± 0.1 (27)	–	1.6 ± 0.1	3.2 ± 0.3 (35)	1.8 ± 0.1
αP192E/βWT	2.4 ± 0.3 (9)	ns	1.5 ± 0.1	3.1 ± 0.7 (15)	1.7 ± 0.3
αP192M/βWT	3.5 ± 0.2 (6)	*	1.6 ± 0.1	3.2 ± 0.6 (11)	1.4 ± 0.2
αP192Y/βWT	1.7 ± 0.4 (5)	ns	1.5 ± 0.1	2.1 ± 0.3 (6)	1.2 ± 0.2
αY194R/βWT	1.7 ± 0.2 (7)	ns	1.5 ± 0.1	5.9 ± 2.3 (6)	2.1 ± 0.5
αSSS^d^→GSD/βWT	1.2 ± 0.2 (10)	ns	1.3 ± 0.1	2.9 ± 0.6 (7)	1.8 ± 0.4
αE273R/βWT	6.3 ± 1.1 (9)	**	1.5 ± 0.1	2.2 ± 0.5 (9)	1.0 ± 0.2
αF276A/βWT	9.3 ± 0.9 (7)	**	1.3 ± 0.1	3.1 ± 0.5 (15)	0.2 ± 0.03
αF276W/βWT	5.5 ± 0.6 (9)	**	1.5 ± 0.1	3.0 ± 0.4 (19)	1.2 ± 0.2

EC_50_, half-maximal effective concentration of Glu. *n*_H_, Hill coefficient of activation by Glu. Number of determinations is provided in parentheses. ^a^Same cells as used for the EC_50_. ^b^Same cells as used for the amplitudes obtained by EC_50_ concentrations (conc.) of Glu. ^c^Not determined due to very small currents recorded in response to 10 mM Glu. ^d^Positions 236–238. Data are mean ± SEM. ANOVA, one-way analysis of variance performed with Dunnett’s multiple comparison test for the EC_50_ values. Asterisks indicate a statistically significant difference in comparison with the GluClαWT/βWT receptor, with probabilities of *0.01 < *P* < 0.05 and **0.001 < *P* < 0.01; ns, does not differ significantly from the GluClαWT/βWT receptor (*P* > 0.05). Same ANOVA analysis for the *n*_H_ values indicates no significant difference between the various mutant and wild type receptors (*P* > 0.05), except for the αSSS→GSD/βWT and αF276A/βWT mutants where *P* < 0.05.

### Effects of Site-Specific Mutations on the Sensitivity of the Heteromeric GluClR to Glu

Previous functional studies with various Cys-loop receptor mutants have shown that the M2-M3 loop is involved in the gating process (Campos-Caro et al., 1996; Lynch et al., 1997; Boileau and Czajkowski, 1999; Grosman et al., 2000; Bera et al., 2002; Absalom et al., 2003; Kash et al., 2003; Bouzat et al., 2004, 2008; Grutter et al., 2005; Law et al., 2005; Lee and Sine, 2005; Lummis et al., 2005; Reeves et al., 2005; Sala et al., 2005; Xiu et al., 2005; Jansen and Akabas, 2006; Jha et al., 2007; Lee et al., 2008, 2009; Chang et al., 2009; Paulsen et al., 2009; Perkins et al., 2009; Pless and Lynch, 2009b; Wiltfong and Jansen, 2009; Yamodo et al., 2010; Hamouda et al., 2011; Zhang et al., 2011, 2013; Dellisanti et al., 2013; Mnatsakanyan and Jansen, 2013; Scott et al., 2015; Bertozzi et al., 2016). Hence, in this study we refrained from substituting amino acids in the M2-M3 loop or amino acids that, according to the X-ray crystal structure of the GluClα_cryst_R, might directly contribute to the bond network of the β1β2, Cys and β8β9 loops with the M2-M3 loop (Hibbs and Gouaux, 2011). Instead, following a careful inspection of the 3-D structure of the GluClα_cryst_R (Hibbs and Gouaux, 2011), we mutated residues in the full-length GluClα subunit that were expected to be involved in intersubunit contacts between the Cys and β8β9 loops (Figure 1B and Table 1). We also mutated residues in the pre-M1 and M1 regions that might have direct contacts with the M2-M3 loop of the adjacent subunit (Figures 1B,C and Table 1).

**Figure 3:**
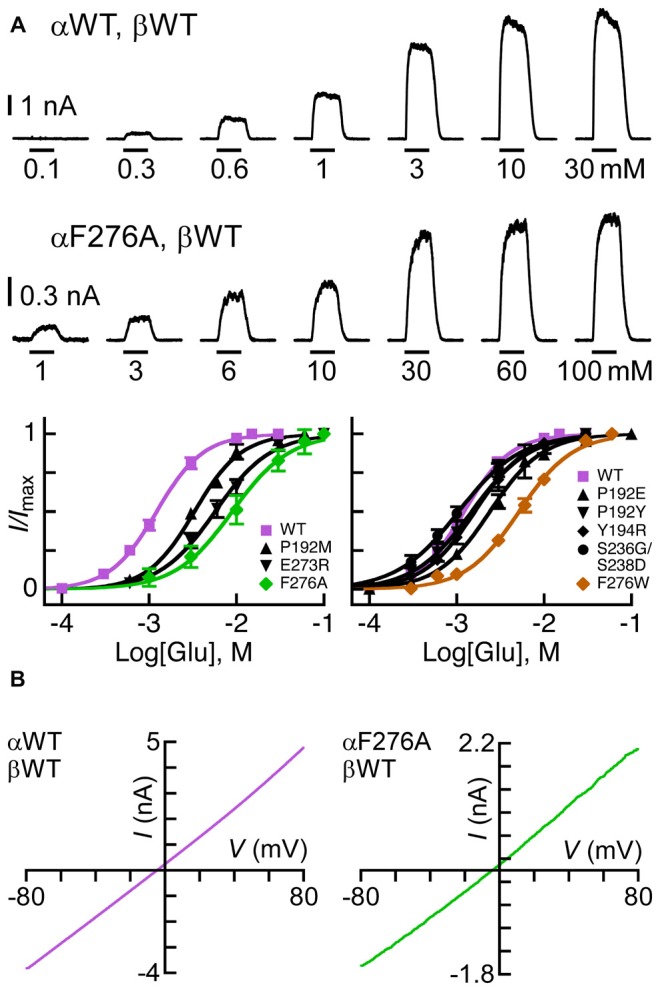
**Sensitivity of GluClα/β receptors to Glu. (A)** Representative current traces measured in cells co-transfected with the indicated subunits (two upper rows). Horizontal bars correspond to 1-s applications of Glu in millimolar concentrations as indicated below the bars. Recordings were performed at +60 mV. The lowest row of this panel shows Glu dose-response curves for receptors assembled from the GluClα subunits indicated in the insets and the GluClβWT subunit. Curves were fitted to the averaged data points with a nonlinear regression using the Hill equation (Equation 1) (*r*^2^ > 0.99). Error bars correspond to SEM. **(B)** Current-voltage (*I/V*) relations obtained upon the application of Glu-EC_50_ concentrations over a voltage ramp lasting 250 ms in cells expressing the indicated subunits (see “Materials and Methods” Section). The *I*_+60 mV_/*I*_-60 mV_ ratios calculated for the GluClαWT/βWT and the GluClαF276A/βWT receptors are 1.3 ± 0.03 and 1.44 ± 0.1 (mean ± SEM), respectively; *P* = 0.14 for three determinations each.

According to the GluClα_cryst_R structure (PDB code 3RIF), αP192 and αY194 of the Cys loop form van der Waals interactions across the intersubunit α/α interface with αS237 of the β8β9 loop in the neighboring subunit (Hibbs and Gouaux, 2011; Figure 1B; see Table 1 for numbering in the GluClα_cryst_ subunit). Replacement of αP192 by either E, M or Y did not substantially change the EC_50_ and Hill coefficient (*n*_H_) for Glu (Figure 3A and Table 2), which may indicate that the contact at this position is not important or it is preserved by the substituting amino acids. Furthermore, replacing αY194 by the homologous residue of the GluClβ subunit created a GluClαY194R/βWT receptor that displays wild type behavior in terms of the EC_50_ and Hill coefficient for Glu (Figure 3A and Table 2).

As said, αS237 of the β8β9 loop interacts with the αP192 and αY194 of the Cys loop of the adjacent subunit. So, we wished to assess whether the sequence difference between the α and β subunits (αSS^237^S vs. βGS^207^D; Figure 2) imparts different conformation-dependent contact with functional relevance. Hence, we also replaced the αSSS sequence by the homologous βGSD sequence. However, this triple-site substitution exerted no (or minor) effects on the EC_50_ and Hill coefficient of the GluClα[SSS→GSD]/βWT receptor for Glu (Figure 3A and Table 2).

According to the GluClα_cryst_R, αE273 is situated in the pre-M1 linker and its side chain forms van der Waals interactions with αQ243 of the β9 strand and αS332 of the M2-M3 loop in the adjacent subunit (Hibbs and Gouaux, 2011; Figure 1C). Hence, αE273 might play a role in ion-channel gating by transducing movements of the outer β-sheet to the M2-M3 loop of the neighboring subunit. However, only moderate 4.2-fold increase in the Glu-EC_50_ and no change in the Hill coefficient of activation by Glu were observed for the GluClαE273R/βWT receptor (Figure 3A and Table 2). These observations indicate that the charge at position α273 does not play a substantial role in the receptor–channel gating process; otherwise, a larger effect would have arisen.

According to the crystal structure of the homomeric GluClα_cryst_R, αF276 is located in the upper helical turn of the first transmembrane helix (M1) close to the IVM binding pocket, but it does not have any contacts with IVM (Figure 1B). In the GluClα_cryst_R, αF276 forms van der Waals interactions with αP330 located in the M2-M3 loop of the neighboring subunit (Hibbs and Gouaux, 2011; Figure 1B). Changing the bulky hydrophobic Phe at position α276 to the small hydrophobic alanine residue increased the Glu-EC_50_ of the GluClαF276A/βWT mutant receptor by 6.2-fold and slightly decreased the Hill coefficient of activation by Glu (Figure 3A and Table 2). In contrast, substituting a very large hydrophobic residue at this position to give the GluClαF276W/βWT mutant receptor increased the Glu-EC_50_ by 3.7-fold, with no substantial change in the Hill coefficient of activation by Glu (Figure 3A and Table 2).

The rationale for using a positive membrane voltage in these experiments was initially based on previous experiments showing that the wild type heteromeric GluClα/β receptor provides an *I/V* curve that weakly rectifies outwardly when heterologically expressed in *Xenopus* oocytes (Cully et al., 1994). As such, the currents at +60 mV were larger by ~4.5 folds than the currents at −60 mV (Cully et al., 1994). Since it was important for us to achieve robust responses at Glu-EC_50_ concentrations when we further determined IVM/Glu peak amplitude ratios (see below), we measured responses at +60 mV. However, here we have used approximately equimolar extra- and intracellular chloride ion concentrations (150.8 mM and 138 mM, respectively), whereas the extracellular and calculated intracellular chloride-ion concentrations used in the *Xenopus* oocyte system were 122.6 mM and 33 mM, respectively (Cully et al., 1994). Accordingly, here, the currents measured at +60 mV are larger by only ~1.4 folds than the currents measured at −60 mV for both, the GluClαWT/βWT and the mutant GluClαF276A/βWT receptors that display close-to-linear *I/V* relations (Figure 3B). It should therefore be emphasized that, hereafter, the behavior of the WT and mutant receptors were compared under exactly the same experimental conditions.

### Sensitivity of Heteromeric GluClR Mutants to IVM Relatively to their Responsiveness to Glu-EC_50_ Concentrations

**Figure 4:**
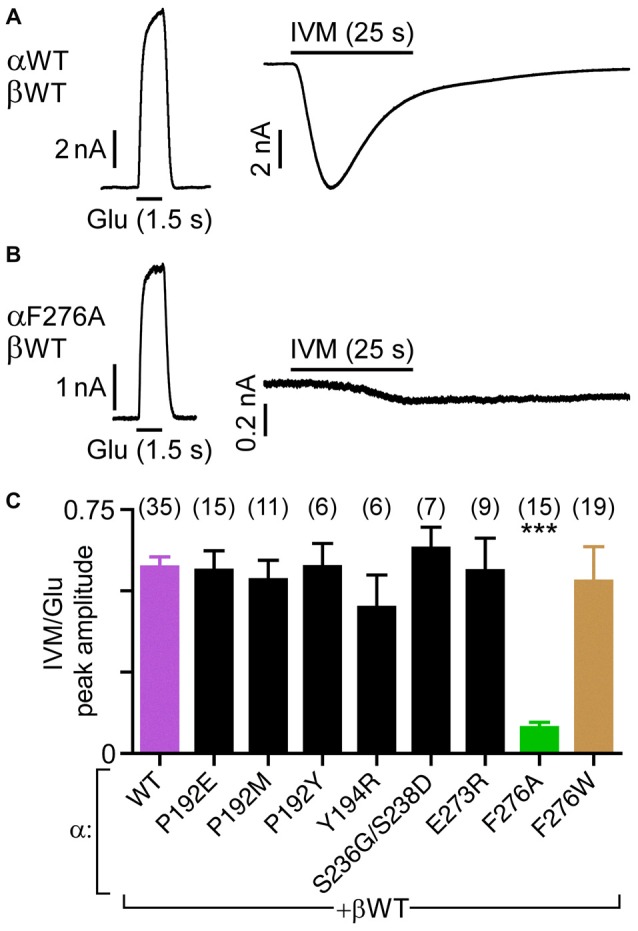
**Sensitivity of GluClα/β receptors to IVM relatively to their responsiveness to Glu-EC_50_ concentrations. (A,B)** Representative current traces elicited in response to EC_50_ concentrations of Glu (left, +60 mV) and 500 nM IVM (right, −60 mV). Cells were co-transfected with the indicated subunits. **(C)** Histogram corresponding to the ratio of IVM-elicited over Glu-elicited current peak amplitudes. EC_50_ concentrations of Glu (Table 2) and 500 nM IVM were used. Cells were co-transfected with the GluClα subunits indicated below the bar graph together with the GluClβWT subunit. ***P < 0.0001; no statistical difference was observed between the other mutants and the wild type receptor (*P* > 0.06). The number of cells is indicated in parentheses above the graph’s bars.

To compare between the sensitivities of the wild type and mutant GluClα/β receptors to IVM, we used the Glu-EC_50_ concentrations and analyzed the relative IVM/Glu responses. To this end, only cells that showed robust responses to Glu at +60 mV, were subsequently challenged by 500 nM IVM (e.g., Figures 4A,B). As such, we ascertained that weak responses to IVM are not due to low expression levels; rather they might reflect a reduced receptor sensitivity to IVM (e.g., Figure 4B). Yet, determination of IVM-EC_50_ values were required for mutants showing reduced IVM/Glu response ratio (see further below). The responses to IVM were measured at −60 mV, a membrane voltage that keeps the cell stable for a long time application. Then, in each cell, the peak current obtained upon IVM application was divided by the peak current obtained upon Glu application. As such, variability that could have emerged due to differences in receptor expression levels was avoided.

Figure 4B shows that the heteromeric GluClαF276A/βWT receptor robustly responds to the Glu-EC_50_ concentration, but weakly responds to IVM (500 nM). Compared to the GluClαWT/βWT receptor, the IVM/Glu response ratio of the GluClαF276A/βWT receptor decreased by ~7-fold (Figure 4C, purple and green bars, respectively). All other mutations in the GluClα subunit had no statistically significant effect on the sensitivity to IVM, as determined relatively to their responsiveness to Glu-EC_50_ concentrations (Figure 4C).

The current shown in Figure 4A (right) declines in the presence of IVM, despite that IVM stabilizes an open channel conformation. It should therefore be noted that previous studies demonstrated that the decline of the inward current (outflow of Cl^−^ ions) conveyed by heteromeric GluClα/βRs is due to a decrease in the electrochemical driving force acting on the chloride ions during the time window of the recording (Slimko et al., 2002; Degani-Katzav et al., 2016).

### Potentiation by IVM of the GluClαF276A/βWT Receptor’s Responses to Glu

Replacing the bulky αF276 in the tip of M1 by the much smaller side chain of alanine (GluClαF276A/βWT receptor) can be envisioned to disrupt the multiple van der Waals contacts that the native Phe had with P330 of the M2-M3 loop in the neighboring subunit (Figure 1B). So, as a result, it is possible that M1, M2 and M3 dislocate one with respect to another, which could make the receptor opening process more difficult. Since IVM binds at the intersubunit interface and makes extensive contacts with the M1 and M3 of adjacent subunits (Hibbs and Gouaux, 2011), we examined how the drug affects the macroscopic activation of the GluClαF276A/βWT mutant receptor by Glu.

**Figure 5:**
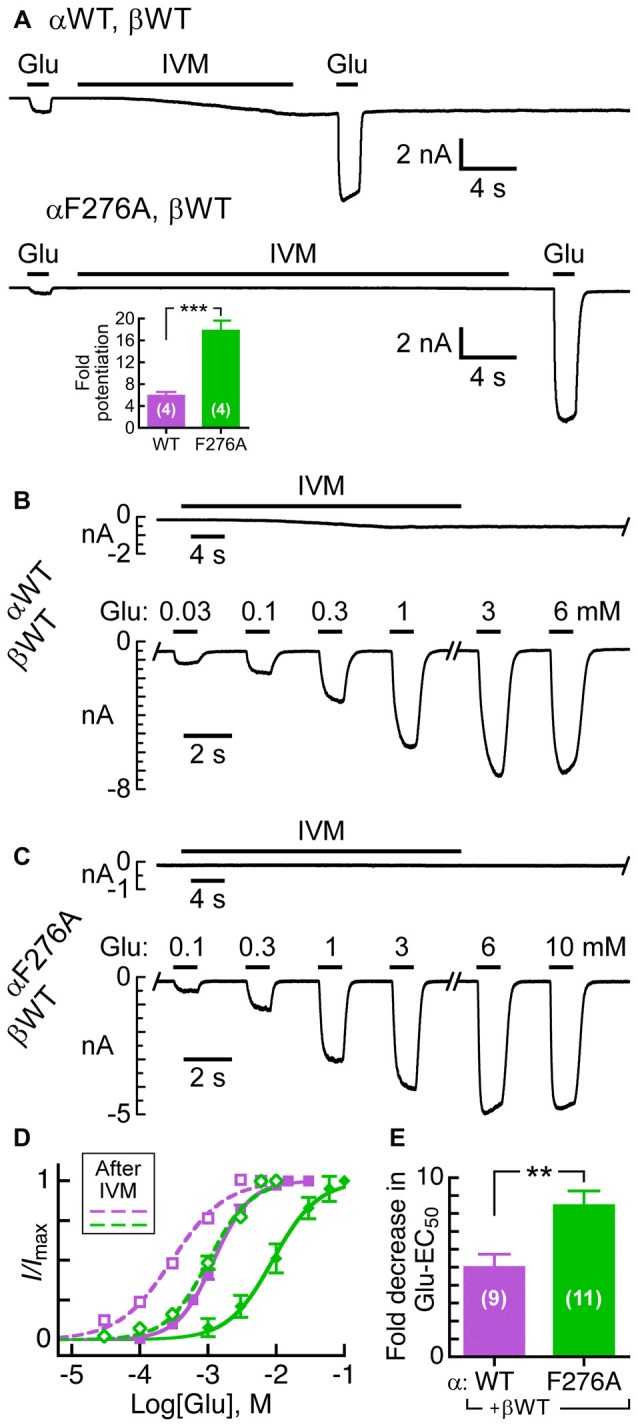
**Effect of pre-exposure to IVM on the activation of GluClα/β receptors by Glu. (A)** Representative current traces of the potentiation effect exerted by IVM on Glu-elicited responses in cells co-transfected with the indicated subunits. Glu concentrations before and after IVM application: 0.3 mM (upper trace); 1 mM (lower trace). IVM concentrations: 7 nM (upper trace); 50 nM (lower trace). Supplementary Figure S1 shows magnification of the lower trace. Inset, fold-potentiation for the GluClαWT/βWT (5.9 ± 0.6) and GluClαF276A/βWT (17.9 ± 1.8) receptors. Data are mean ± SEM. The number of determinations is indicated in white; ***P < 0.001. **(B,C)** Representative current traces elicited by increasing Glu concentrations after IVM pre-application. The time of delay between the end of IVM application and the beginning of Glu application was 20 s. IVM concentrations, as in **(A)**. Oblique lettering indicate the expressed subunits. **(D)** Glu dose-response curves for experiments exemplified in **(B,C)**. Dashed curves correspond to measurements performed after pre-exposure to IVM in cells expressing the GluClαWT/βWT (purple) or GluClαF276A/βWT (green) receptors. Curves were fitted as in Figure 3A (*r*^2^ > 0.98). Error bars correspond to SEM. Continuous curves correspond to measurements performed without pre-exposure to IVM (taken from Figure 3A). Glu-EC_50_ after pre-exposure to IVM: 0.3 ± 0.03 mM for the GluClαWT/βWT receptor, and 1.1 ± 0.1 mM for the GluClαF276A/βWT receptor (*P* < 0.0001). Hill coefficients of activation by Glu for the WT and mutant receptors (dashed curves): 1.2 ± 0.07 and 1.5 ± 0.03, respectively (*P* < 0.003). Statistical significance for the Hill coefficients before vs. after exposure to IVM: GluClαWT/βWT receptor, *P* < 0.001; and GluClαF276A/βWT receptor, *P* < 0.04. **(E)** Fold decrease in Glu-EC_50_ observed after pre-exposure to IVM. Data in **(D,E)** are mean ± SEM; number of determinations in white. ** 0.001 < *P* < 0.005.

It was previously shown that 5 nM IVM potentiates the Glu-sensitive currents of the wild type GluClα/βR by ~5-fold, as measured in *Xenopus* oocytes (Cully et al., 1994). For an appropriate reference in CHO cells, we first determined the capacity of IVM to potentiate the response of the wild type heteromeric receptor to Glu. To this end, 0.3 mM Glu was initially applied to obtain a weak current response (Figure 5A, upper trace, leftmost response). This Glu concentration activates ~7% of the GluClαWT/βWT receptor population, as can be calculated based on the Glu dose-response curve (Figure 3A). This weak response increased by ~6-fold when 0.3 mM Glu was applied again shortly after exposure of the cell to 7 nM IVM (Figure 5A, upper trace and the inset). The same application protocol was employed for the mutant GluClαF276A/βWT receptor with two exceptions. First, we used 1 mM Glu, which activates ~5% of the mutant receptor population. Second, we used 50 nM IVM that is ~7 times the IVM concentration used for the GluClαWT/βWT receptor. This IVM concentration reflects the ~7-fold decrease in the IVM/Glu response ratio of the GluClαF276A/βWT mutant receptor (Figure 4C). Figure 5A (lower trace and the inset) shows that in the GluClαF276A/βWT receptor, IVM potentiated the response to Glu by ~18-fold. Notably, the response of the GluClαF276A/βWT receptor to IVM was very weak, but could clearly be visualized upon magnification (see Supplementary Figure S1).

In order to quantify the potentiation effect of IVM more accurately, we first exposed the expressing cell to the low IVM concentrations mentioned in Figure 5A, and as soon as the IVM-elicited current reached to the steady state, we challenged the expressing cell with increasing Glu concentrations (e.g., Figures 5B,C). The corresponding Glu dose-response curves are shown in Figure 5D (EC_50_ and *n*_H_ values are detailed in the legend). Evidently, in both the wild type and mutant receptors the Glu dose-response curves have shifted to the left (dashed lines) due to the pre-application of IVM (Figure 5D). In addition, after pre-exposure to IVM, the Hill coefficient for Glu slightly increased in the case of the mutant GluClαF276A/βWT receptor, whereas it slightly decreased in the case of the GluClαWT/βWT receptor (Figure 5D and its legend). Figure 5E indicates that the IVM-induced decrease in Glu-EC_50_ is ~5-fold and ~8.5-fold for the GluClαWT/βWT and GluClαF276A/βWT receptors, respectively; despite that the mutant receptor displayed weaker responsiveness to IVM than the wild type receptor (e.g., Figure 5A, lower trace vs. upper trace; Figure 5C vs. Figure 5B).

### Concentration-Response Relationships Indicate that αF276 is Important for IVM Accommodation

**Figure 6:**
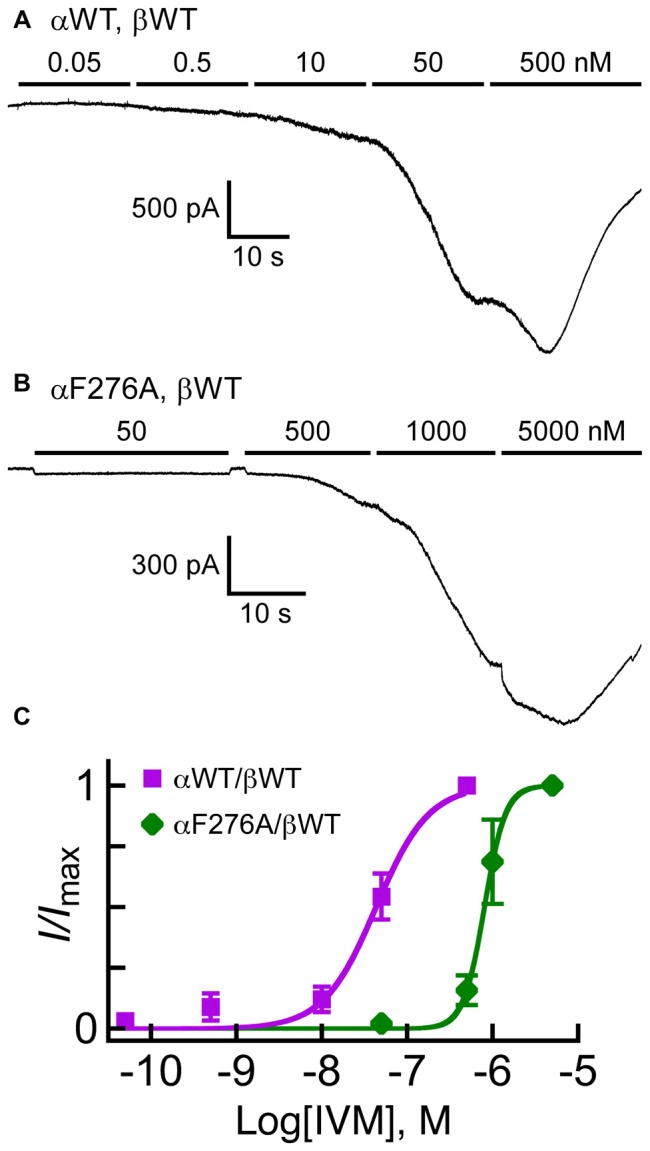
**Responses of GluClα/β receptors to cumulative concentrations of IVM. (A,B)** Representative current traces measured in cells co-transfected with the indicated subunits. Horizontal bars correspond to applications of IVM in increasing nanomolar concentrations, as indicated above the bars. Recordings were performed at −60 mV. **(C)** IVM dose-response curves for experiments exemplified in **(A,B)**. The Curves were fitted to the averaged data points with a nonlinear regression using the Hill equation (Equation 1) (*r*^2^ > 0.98). Error bars correspond to SEM. IVM-EC_50_ values for the GluClαWT/βWT and GluClαF276A/βWT receptors are 40 ± 10 nM and 802 ± 170 nM, respectively (mean ± SEM of six determinations for each receptor type; *P* = 0.01). Hill coefficients of activation by IVM for the GluClαWT/βWT and GluClαF276A/βWT receptors are 1.5 ± 0.2 and 3.5 ± 0.40, respectively (mean ± SEM; *P* = 0.001).

The results presented in the previous sections may suggest that the αF276A mutation increases the Glu-EC_50_ value by affecting allosterically the conformation of the Glu-binding pockets and thereby changing the mode of Glu accommodation. However, another possibility is that the channel has become generally less easy to open with no essential change in the mode of Glu binding, while IVM retains its positive modulation activity. To understand the impact of the αF276A mutation further, we analyzed the IVM concentration-response relationships for the GluClαWT/βWT and GluClαF276A/βWT receptors. Because IVM currents are irreversible, we established the IVM concentration-response relationships by successively applying increasing IVM concentrations as has previously been performed, for example, in the case of the GlyR (Lynagh and Lynch, 2010; Lynagh et al., 2011). Since the responses of the mutant GluClαF276A/βWT receptor to 500 nM IVM were weak (e.g., Figure 4B, right; Table 2), in these experiments we have used a more powerful transfection reagent and raised the amount of cDNA used in cell transfections (see “Materials and Methods” Section). Figures 6A,B show representative current traces for the GluClαWT/βWT and GluClαF276A/βWT receptors. Such experiments were used to establish the IVM dose-response curves shown in Figure 6C. These dose-response curves indicate that the IVM-EC_50_ of the GluClαWT/βWT receptor (40 nM) was 20-fold lower than that of the GluClαF276A/βWT receptor (802 nM; see statistical analysis in the legend to Figure 6C). The wild type and mutant receptors also differ in their Hill coefficient of activation by IVM, which was found to be 1.5 for the GluClαWT/βWT receptor and 3.5 for the GluClαF276A/βWT mutant receptor (Figure 6C; see statistics in the legend).

## Discussion

In the homomeric GluClα_cryst_R, large portions of the Cys and β8β9 loops are situated at the interface between neighboring subunits, where they directly interact with each other (Hibbs and Gouaux, 2011; Figure 1A). That is, in an α/α intersubunit interface, αP192 and αY194 of the Cys loop form van der Waals interactions with αS237 of the β8β9 loop of the neighboring subunit (Hibbs and Gouaux, 2011; Figure 1B). Potential homologous contacts might also exist in the heteromeric GluClα/βR, at α/β, β/α and α/α intersubunit interfaces. Replacement of residues in this contact region of the GluClα subunit by the homologous residues of the GluClβ subunit (GluClαY194R/βWT and GluClαSSS→GSD/βWT receptors) exerted no substantial effects on the Glu-EC_50_ and the receptor subunits’ cooperativity. These results imply that the Cys–β8β9-loop contacts at this specific point are either preserved in the potential heteromeric intersubunit interfaces (α/β or β/α), or alternatively are not essential for macroscopic activation. Furthermore, the substitution of GluClαP192 for charged, uncharged or polar bulky residues appears not to be detrimental for the conformation of the Cys loop since the Glu-activation macroscopic properties were not essentially changed (Table 2). Likewise, the unchanged IVM/Glu response ratio of the heteromeric GluClRs bearing the αP192E/M/Y and αY194R mutations (Figure 4C) suggests that the conformation of the IVM-binding site is not allosterically affected by these mutations.

According to the GluClα_cryst_ receptor, E273 is located in the pre-M1 region (Hibbs and Gouaux, 2011) that was previously shown to carry amino acids involved in the gating process in other Cys-loop receptors (Boileau and Czajkowski, 1999; Chang et al., 2003; Hu et al., 2003; Kash et al., 2004; Lee and Sine, 2005; Xiu et al., 2005; Keramidas et al., 2006; Price et al., 2007; Purohit and Auerbach, 2007; Wang et al., 2007; Mercado and Czajkowski, 2008; Cederholm et al., 2009; Lee et al., 2009; Mukhtasimova et al., 2009; Pless and Lynch, 2009a; Bruhova and Auerbach, 2010; Hanson and Czajkowski, 2011; Pless et al., 2011; Wang and Lynch, 2011; Gonzalez-Gutierrez et al., 2013; Mukhtasimova and Sine, 2013; Shen et al., 2016). In the GluClα_cryst_R, αE273 is sandwiched between Q243 located in the β9 strand of the same subunit and S332 that is located in the M2-M3 loop of the adjacent subunit (Hibbs and Gouaux, 2011; Figure 1C). More particularly, the carboxylic oxygens O_ε1_ and O_ε2_ of E273 are located at distances of 3.4 and 3.8 angstroms from the C_β_ atoms of S332 and Q243, respectively; with no seemingly close opposite charge to pair with. As such, in the homomeric GluClα_cryst_R, E273 probably makes van der Waals interactions with these two residues. Furthermore, it can be envisioned that upon a slight motion of the outer β-sheet (which β9 belongs to), the carboxylic oxygens of E273 could become sufficiently close to form hydrogen bonds with Q243 and S332. Hence, based on the GluClα_cryst_R structure (Hibbs and Gouaux, 2011), E273 could be involved in transduction of neurotransmitter-binding energy to the channel gate via the M2-M3 loop. Yet, it appears here that the charge at this position does not have a fundamental role in such a process as the αE273R mutation increased the Glu-EC_50_ by only 4.2-fold. Hence, the moderate effect of this mutation may be attributed to a slight change in the van der Waals (or potential hydrogen) bond network that the replaced (native) residue at this position is involved in.

These observations were quite surprising for us because in various other Cys-loop receptors the residue at the homologous position does play a role in the gating process, despite that it is not conserved. For example, in the mouse 5HT_3A_R, when R245 (the homologous position of GluClαE273; Figure 2) was mutated to A or E, larger impacts on the receptor function have been observed (Hu et al., 2003; Price et al., 2007). This arginine of the mouse 5HT_3A_R was suggested to pair ionically with a glutamate residue located on the β9 strand (homologous to GluClαQ243), so as to transduce agonist binding to channel gating (Price et al., 2007). Mutations introduced at the homologous position in the mouse nAChR α1 subunit (L230 in Figure 2) increased the gating equilibrium constant relatively to the wild type receptor (Purohit and Auerbach, 2007). Mutations were also introduced at the homologous position in the human nAChR α1 subunit (L255 in Figure 2). The latter indicated the existence of energetic coupling between this leucine from the pre-M1 region, αF180 and αF182 from the Cys-loop, and αL318 from the M2–M3 loop (amino acid numbering according to Figure 2; Lee et al., 2009). So, it was suggested that the β1-β2 and Cys loops bridge the pre-M1 region and M2-M3 loop to transduce agonist binding into channel gating (Lee et al., 2009). Taken together, in relation to this position, it appears that the invertebrate GluClα/βR differs from the aforementioned vertebrate Cys-loop receptors, since its α273 position plays a minor role in gating. Notably, the αE273R mutation did not change the IVM/Glu response ratio (Figure 4C) although, according to the GluClα_cryst_R structure, E273 interacts with S332 of the M2-M3 loop—very close to I334 that makes a contact with IVM.

EC_50_ depends both on the ligand-binding affinity and efficacy of gating (Colquhoun and Farrant, 1993). Since the 20-fold increase in IVM-EC_50_ of the GluClαF276A/βWT mutant receptor (Figure 6) was accompanied by moderate 6.2-fold increase in its Glu-EC_50_ (Table 2), we suggest that the αF276A mutation reduced the efficacy of channel gating with likely no allosteric effect on the Glu-binding affinity. This suggestion is also supported by the observation that the αF276A mutation caused a slight change in the Hill coefficient of activation by Glu (Table 2), but dramatically changed the Hill coefficient of activation by IVM (Figure 6). An inevitable question therefore emerges; why would the sensitivity to IVM decrease significantly due to a mutation of a residue that does not interact directly with IVM (at least according to the 3-D structure of the GluClα_cryst_ receptor)?

The mutation in the heteromeric GluClαF276A/βWT receptor is located in the first helical turn of M1, outside but close to the IVM-binding pocket that is located between M1 and M3 of neighboring subunits. According to the GluClα_cryst_R 3-D structure, the side chain of the native amino acid (αF276) forms multiple van der Waals interactions with αP330 of the M2-M3 loop of the neighboring subunit (Hibbs and Gouaux, 2011; Figure 1B). αP330 is not conserved throughout Cys-loop receptors, but the GluClβ subunit also has a proline residue at the homologous position in its M2-M3 loop (Figure 2). Therefore, substituting GluClαF276 for an alanine would probably eliminate the Phe–Pro interactions at potential α/α and β/α intersubunit interfaces in the heteromeric GluClαF276A/βWT receptor. Such elimination might enable more freedom for M1 to move relatively to M3 and thereby could change the position of amino acids that interact with IVM. This interpretation is supported by two sets of experiments. First, in a screen for the sensitivity of the various receptors to IVM relatively to their responsiveness to Glu-EC_50_ concentrations, the ratio of IVM/Glu peak amplitudes was found to be significantly lower for the GluClαF276A/βWT mutant receptor than for the wild type and the other mutant receptors (Figure 4C). Second, independently of the responsiveness to Glu, determinations of IVM-EC_50_ values indicate that the mutant GluClαF276A/βWT receptor is much less sensitive to IVM than the GluClαWT/βWT receptor (as discussed above). Furthermore, the wild type and mutant receptors greatly differ in their Hill coefficients of activation by IVM (*n*_H_ = 1.5 and 3.5 respectively; Figure 6C). It therefore appears that the binding of two IVM molecules is required to achieve full macroscopic activation of the GluClαWT/βWT receptor, whereas the binding of at least three IVM molecules is required to fully activate the mutant GluClαF276A/βWT receptor. Taken together, we suggest that αF276 plays an important role in IVM accommodation because it interacts with the M2-M3 loop and thereby contributes to the stabilization of the IVM-binding pocket between M1 and M3 of adjacent subunits.

The loss of a contact between the tip of M1 and the M2-M3 loop might possibly dislocate M1, M2 and M3 and thereby reduce the channel-gating efficacy. If this is actually the case in the GluClαF276A/βWT mutant receptor, then constraining M1 in respect to M3 by IVM is anticipated to improve the efficacy of channel gating. Indeed, despite that the GluClαF276A/βWT mutant receptor is 20-fold less sensitive to IVM than the GluClαWT/βWT receptor, it was sufficient to increase the pre-applied IVM concentration only by ~7 times, in order to get larger potentiation of Glu currents in the mutant receptor (~18-fold) than in the wild type receptor (~6-fold; Figure 5A, inset). Moreover, an increase of the pre-applied IVM concentration by ~7-fold improved (reduced) the Glu-EC_50_ of the mutant receptor to a larger fold-extent than in the WT receptor (~8.5-fold vs. 5-fold, respectively; Figures 5D,E). Notably, those differences in IVM potentiation were obtained even though the pre-applied IVM activated the mutant to lesser extent than the wild type receptor (Figure 5). Taken together, the capability of IVM to bridge between M1 and M3 of adjacent subunits, likely by forming multiple interactions with these transmembrane segments, largely compensates for the reduction in channel-gating efficacy. We suggest that the reduction in channel-gating efficacy is most likely due to the loss of the aforementioned interaction between the tip of M1 and the M2-M3 loop in the mutant receptor. We further hypothesize that, in the presence of a subthreshold IVM concentration, M1 and M3 of the mutant receptor likely adopts WT-like conformation that is typical of a preopen state sensitive to Glu.

Noteworthy, in comparison with the effect of the αF276A mutation, the αF276W mutation exerted weaker effect on the Glu-EC_50_, no effect on the Hill coefficient for Glu (Figure 3A and Table 2), and no effect on the IVM/Glu response ratio (Figure 4C). We therefore suggest that a tryptophan residue at position α276 interacts with the M2-M3 loop of the neighboring subunit akin to the native phenylalanine.

It is also noteworthy that, based on previous functional studies, motions of M1 and M3 were suggested to take place during activation in other Cys-loop receptors. Using cysteine substitutions and disulfide crosslinking experiments with a GABA_A_R, demonstrated that the extracellular ends of M1 and M3 of the adjacent α1 and β2 subunits get closer to each other upon activation (Bali et al., 2009). Other functional studies showed that, the susceptibility of amino acids in M1 to various chemical modifications is changed following the transition of resting ACh- and GABA-gated Cys-loop receptors to their active state (Akabas and Karlin, 1995; Yu et al., 2003; Arevalo et al., 2005; Li et al., 2006; Pandhare et al., 2012). It is of interest to note that IVM activates mammalian GABA-gated chloride channels as well (Williams and Risley, 1982; Olsen and Snowman, 1985; Sigel and Baur, 1987; Krůsek and Zemková, 1994; Adelsberger et al., 2000; Lynagh and Lynch, 2012b; Ménez et al., 2012), possibly by binding to a pocket between M1 and M3 of adjacent subunits akin to the GluClRs. So, this potential IVM-binding pocket might overlap the well-characterized binding site of GABA_A_Rs for the intravenous anesthetic agent etomidate (Li et al., 2006, 2010; Olsen and Li, 2011; Chiara et al., 2012; Stewart et al., 2013a,b, reviewed in Olsen et al., 2014). Hence, the current study might be relevant to further research that aims at better understanding of how certain general anesthetics modulate the activity of GABA-gated Cys-loop receptors.

## Author Contributions

ND-K, RG, MW and YP designed the research, performed the research and analyzed the data. ND-K and YP wrote the article.

## Conflict of Interest Statement

The authors declare that the research was conducted in the absence of any commercial or financial relationships that could be construed as a potential conflict of interest.
