# Supplementary material for: Mutational Analysis at Intersubunit Interfaces of an Anionic Glutamate Receptor Reveals a Key Interaction Important for Channel Gating by Ivermectin
Source: Front Mol Neurosci. 2017 Apr 6;10:92. doi: 10.3389/fnmol.2017.00092 (PMC5382172; doi:10.3389/fnmol.2017.00092)
Supplement: Supplementary file 1 [file Image_1.pdf]

SUPPLEMENTARY MATERIAL

Mutational analysis at intersubunit interfaces of an anionic glutamate receptor reveals a key interaction important for channel gating by ivermectin

Nurit Degani-Katzav[1], Revital Gortler[1], Marina Weissman[1], and Yoav Paas[1,*]

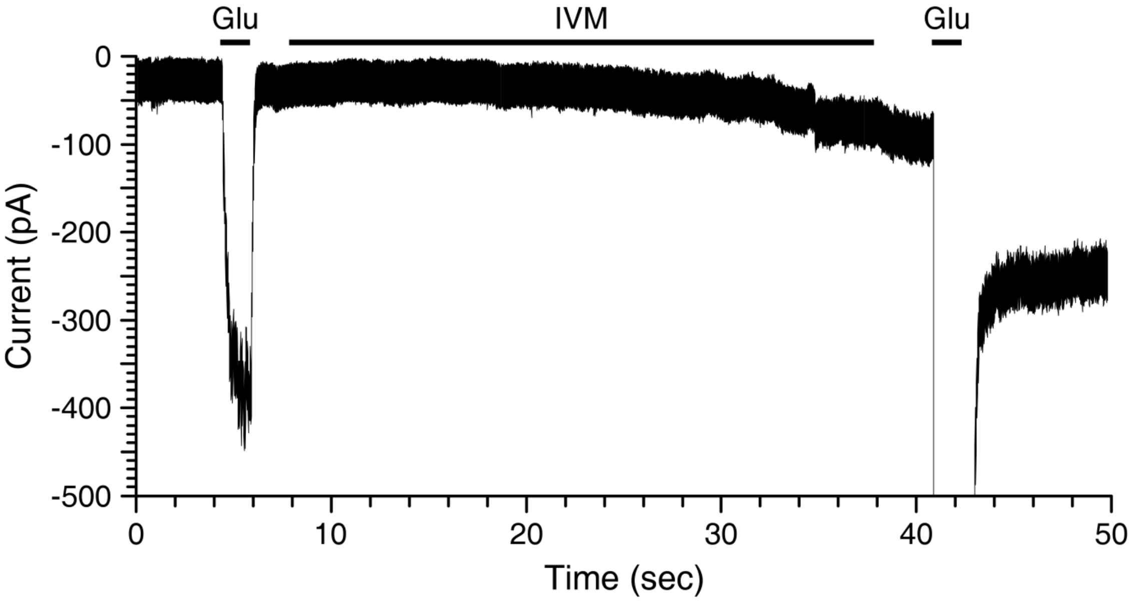

Supplementary Figure S1. Slow inward current is observed upon IVM application (50 nM) to a cell expressing the GluClαF276A/βWT receptor. The magnified trace corresponds to the lower current trace of Figure 5A. It shows the weak effect of IVM on chloride currents through the mutant receptor. The magnitude of the IVM-elicited current, as measured just before the second application of Glu, was ~70 pA. Measurements were performed at –60 mV.
